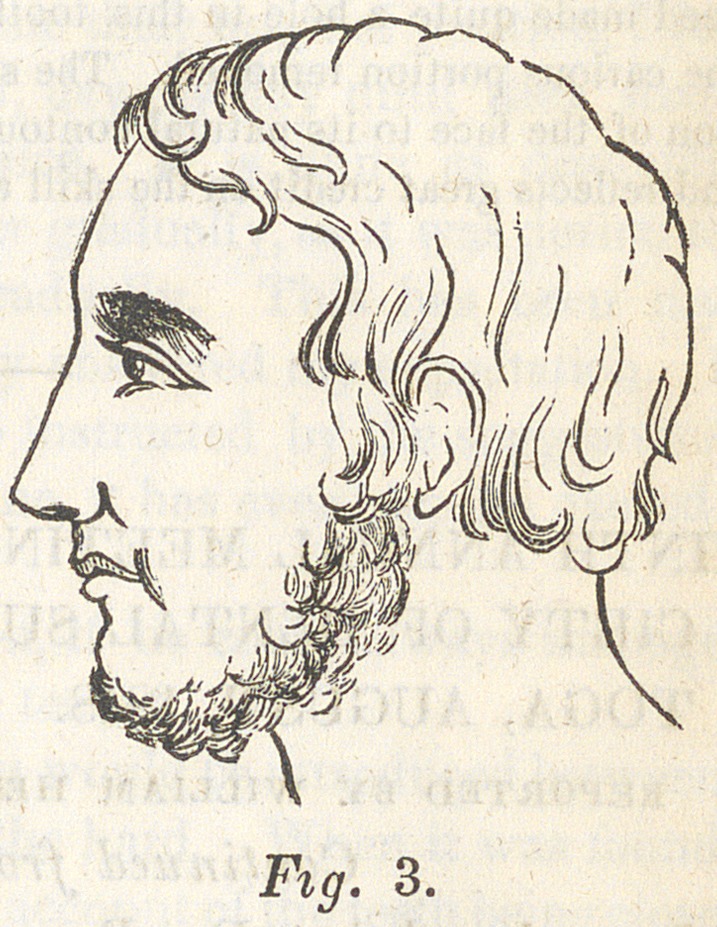# Dr. Sanford’s Letter

**Published:** 1851-01

**Authors:** 


### DR. SANFORD'S LETTER.

CHILLICOTHE, DEC. 27th, 1850.

*Prof. James Taylor:*

DEAR SIR:—The following case occurred in my practice some two years since, and if you deem it of sufficient importance, you can give it a place in your excellent journal.

Mr. J. W. D. of Hardy county, Va., called on me for advice in the winter of 1848, in reference to a great deformity of the upper jaw—having an appearance of an enlargement of the alveolar processes, corresponding to the left central incisor, left cuspidatus, and dens sapientia. These portions of the maxillary bone projected very much, causing great protrusion of the superior lip.

Fig. 1 on the opposite page gives a very correct representation of the patient's face, when I first made an examination of the case.

The three above named teeth were absent, and being undecided as to the nature of the enlargement, I made an incision over the enlarged process, and found no indication of disease in the bone itself.

I then took a small hand drill, and by two or three revolutions of the same, the point of the instrument came in contact with a very hard substance, which I at once pronounced a tooth; but notwithstanding an assurance on my part, in answer to an interrogatory of Mr. D., that I could by an operation entirely remove the deformity, yet he declined having it undertaken at that time.

*Fig. 2.*

About one year after this, he however called upon me again, and requested me to operate, which I immediately proceeded to do. I commenced the operation by making a deep incision on each side of the frænum labiorum, which I carried up to the nasal fossa. I then took a chisel-shaped instrument and cut away the external portion of the enlarged alveolar processes, which to my surprise exposed the three absent teeth already named, which I found embedded in the bone, and occupying the relative parts to each other, as represented in the drawing fig. 2.

I succeeded in removing the teeth without any difficulty by the use of the forcep. There was no hemorrhage, and I put the patient under the ordinary treatment after operations on the mouth. The wound soon healed, and I had the satisfaction of seeing my patient as represented in fig. 3, four months after the operation.

*Fig.* 1.

*Fig.* 3.

Mr. D.'s mouth would to most persons present the appearance of containing a full set of teeth. The alveolar arch having contracted so that the space these teeth would have filled is in a measure supplied by the approximation of the others. One, however, acquainted with the anatomical relations of the mouth, could readily see that these teeth were absent.

Without speculating as to the cause of this anomaly, I will merely allude to the fact connected with the previous history of the case. Mr. D. informs me that when about five years of age he received a violent blow on the mouth and left side of the face. To this circumstance he attributes the origin of the deformity and the displacement of the teeth. I also ascertained on examination of the mouth, that the superior maxilla had been fractured, and that the superior teeth on the left side stood about half an inch on the inside of the inferior teeth. The other teeth are all healthy.

—

The wood cuts which accompany this article, show first the patient betore being operated upon; second, the condition of the three teeth when exposed by the removal of the bone which covered them—the apex of the root of the canine tooth, resting under the nasal fossae, near the medial line. The point of the tooth pressing against the crown of the incisor, which has been broken at its neck, and re-united by the deposit of ossific matter, and the apex of its root resting on the crown of the dens sapientia. Having indeed made quite a hole in this tooth, resembling very much a decay with the carious portion removed. The success of this operation in the restoration of the face to its natural contour has been as we judge quite perfect, and reflects great credit on the skill and judgment of Dr. Sanford.—Ed.

## Figures and Tables

**Fig. 2. f1:**
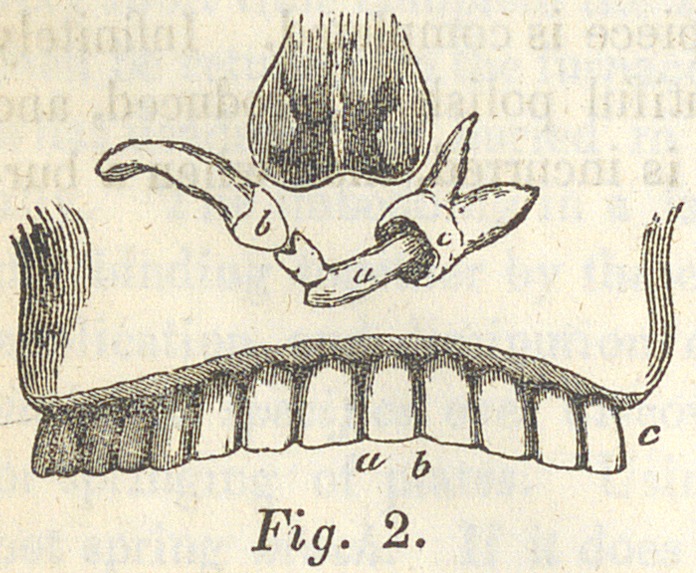


**Fig. 1. f2:**
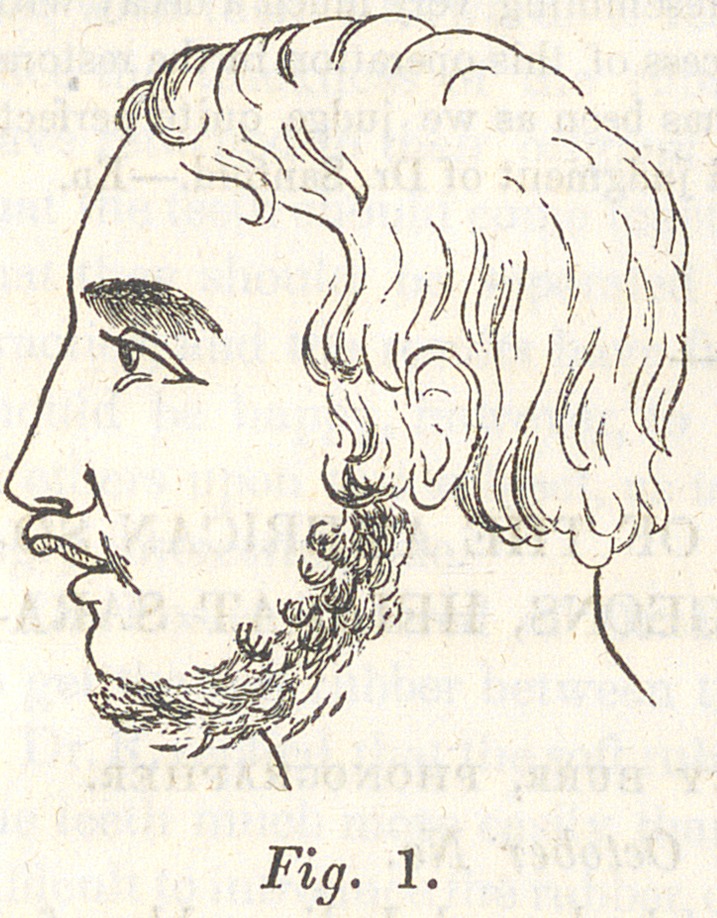


**Fig. 3. f3:**